# Minor Ailments in Older Adults: A Study of Prevalence and Health-Related Behaviors

**DOI:** 10.1177/21501319251398730

**Published:** 2025-12-01

**Authors:** Jeffrey Taylor, Parinaz Amirimoghadam, Erin Yakiwchuk

**Affiliations:** 1 University of Saskatchewan, Saskatoon, Canada

**Keywords:** behavioral health, community health, medications, primary care, pharmacy

## Abstract

**Background:**

With chronic diseases such as diabetes and hypertension garnering much needed attention in health care, minor ailments can go somewhat unnoticed.

**Objective:**

To quantify prevalence, symptom burden, and health-related behaviors relative to 31 minor ailments in older adults.

**Methods:**

A cross-sectional survey was carried out in 1 Canadian province. Participants were asked about symptoms experienced over a 2-week period, the disruption to daily activities they incurred, and treatment actions undertaken.

**Results:**

Three-hundred and fifty-six adults with an average age of 72.8 years completed a questionnaire. Participants reported an average of 6.2 ailments over the time period. Back pain (46.9%), joint pain (44.4%), and insomnia (38.5%) were most common. The vast majority had been an issue for longer than 2 weeks. OTC medicines were the most common treatment choice in 16 situations, while doing nothing/watchful waiting was the main recourse in 13. OTC medication was common for headache (63.2%) and heartburn (61.2%), while doing nothing/watchful waiting was common for tinnitus (80.5%) and loneliness (74.2%). Satisfaction and confidence in self-management were highest for acute conditions like headache and cold sores.

**Conclusion:**

Minor ailments are common and diverse in older adults, with many chronic in nature, and a tendency for proactive self-care.

## Background

Minor ailments are non-serious medical conditions that typically require little or no medical intervention. They generally resolve on their own and can often be managed with self-care options such as home remedies or over-the-counter (OTC) medications.

The types that occur are diverse and common. The top 5 minor illnesses experienced in Canada were muscle aches/pains (49%), cold/flu (42%), headaches (37%), cough (27%), and back pain (26%).^1^ A similar pattern was seen with Americans.^2^ In Britain, the 5 most common were tiredness, headache, muscle aches/pains, sleeping problems, and stiffness in joints, with an average of 5.2 ailments over a 2-week period.^3^ In Canada, most adults experienced at least 1 over 1 year.^4^

Ones such as coughs, minor skin infections, and cold sores seldom have a significant impact on overall health. However, despite their name, the cumulative impact of minor ailments is far from trivial. Most don’t enter the formal healthcare system,^5^ yet millions of medical visits accrue each year for them.^6-8^ In Britain, treatment solely for minor ailments accounted for 20% of total available physician workload.^7^ One-quarter of Canadians experienced colds or flu monthly, costing $625 million annually in doctor visits alone.^6^

How the public respond to minor ailments is important to our understanding of health care. United Kingdom researchers investigated the health behaviors relative to 25 symptoms, ranging from minor to potentially serious conditions.^5^ About half took no action for the symptoms presented, while 12% reported a primary care consultation. The proportion seeking information was highest for more serious symptoms such as fainting and blood in stool, while OTC medicine use was highest for minor symptoms such as headaches and colds.

This study aims to quantify the prevalence and health behaviors associated with 31 common minor ailments over a 2-week period. It will build on the ‘symptom iceberg’ work of McAteer and colleagues,^5,9^ but with a geriatric and minor ailment focus. Due to the sheer number clinicians will attend to in primary care,^10^ this will reinforce which ailments are seen in practice and should add perspective on how older adults are managing such situations. In particular, while the literature typically describes such ailments as minor, the proposed study will examine how persistent they may be. Further, patient confidence in dealing with such symptoms has been underreported.

## Methods

This was a cross-sectional survey of older adults in 1 Canadian province. The CheckMarket sample size calculator^11^ was utilized with the following parameters: population of 40,000 older adults, 95% confidence level, 5% margin of error, and an expected response rate of 10 to 15%. A sample size of 381 respondents was determined. To attain this, an estimated 3000 to 3500 individuals were needed.

Ambulatory residents 65 years of age and older living in Saskatchewan were the target group. Subjects were randomly recruited from the University of Saskatchewan’s Citizens Research Panel, a large group of self-identified people who previously expressed an interest in doing surveys. This was accomplished by a case-selecting computer algorithm that randomly selected names from the master list. Once selected, they were contacted by email. The survey service also partnered with third-party panel vendors (Voxco, Zamplia, and EKOS Research) to help produce a representative sample of older adults. Precedence for using citizen panels to access subjects,^12,13^ including those involving OTC medicines, has been established.^14,15^ A $10 gift card was given to responders who completed a questionnaire. Approval from the university’s Ethics Board was obtained on April 19, 2024. Rather than a diary method which enables responders to list every symptom experienced over a specific period of time,^16^ the approach followed previous research which provided responders with a pre-determined list of common symptoms.^5,9^ Thirty symptoms were deemed to be a workable number, balancing comprehensiveness against responder burden. A tentative list of potential minor ailments was identified from the literature.^1-3,5,7,16-25^ A panel of 6 experts in minor ailments and geriatric care then reviewed the list for relevance as ones commonly experienced by older adults. While chronic conditions such as diabetes and osteoporosis are prevalent in this population, the focus was specific to minor ailments that are frequent.

Interrater reliability (IRR) was employed to evaluate expert agreement, providing insight into some variability in clinical perspectives. Eighteen symptoms garnered 100% agreement, while 7 attained 83.3%. Anxiety/stress was simplified to stress (due to an IRR score of 50%), depression/loneliness was reduced to loneliness, night sweats were excluded, gender-specific conditions (vaginal dryness and erectile dysfunction) were added, while crusting eyelids was reconfigured to Blepharitis. The revisions led to a final list of 29 core symptoms, with vaginal dryness and erectile dysfunction rotating between genders (to reflect the 30th ailment for respective genders), with an overall total of 31.

A series of questions were presented to responders regarding symptoms experienced, their duration, impact on daily life, treatment measures undertaken, satisfaction with treatment, and confidence in their ability to treat/manage symptoms. Precedence for survey items, where they existed, guided item development. Health status was documented as poor, fair, good, very good, and excellent as per government standards.^26^ Treatment measures undertaken for minor ailments are now considered common knowledge for this field, ranging from doing nothing to medical care. Symptom duration was extended beyond times outlined by UK researchers.^9^ The scale to measure disruption to daily life was adapted from previous work,^9,27,28^ but deviated from a 10-point scale used for a telephone survey on minor ailments in Canada.^6^ A 2-week recall period was chosen to minimize recall bias and has been used in similar studies.^3,5,9,29^

A pretest was completed by 10 seniors, whose feedback indicated good readability, clarity in questions, and the time needed to reflect upon symptoms and treatment. A pilot study (20 participants) was conducted, representing about 5% of the intended target sample size of 381. The estimated time for completion was 15 min.

The questionnaire was administered via the Voxco online platform from the first week of October to December 2024. An advanced email notice of the project, a link to the questionnaire, and 3 follow-ups were undertaken.

## Results

A total of 3566 questionnaires were distributed, yielding 402 complete responses for a response rate of 11.3%. To potentially control for responder inattentiveness, an attention-check question was placed at the end of the survey, instructing participants to leave it unanswered. If filled in, an assumption of ‘inattention’ was made. Based on this criterion, 46 were removed, leaving 356 valid responses for analysis. Some responders did not answer all questions and missing values are reflected in the totals.

The sample consisted of slightly more female participants (n = 196, 55.1%) than males (n = 160, 44.9%; Table 1). The mean age was 72.8 (SD = 5.7) years. A slight majority (53.1%) resided in the main towns/cities of the province. Nearly half (44.9%) of participants completed college or university. Many rated their health as good (41.6%) or very good (33.1%).

**Table 1.** Socio-demographic Details of Participants.

Demographic characteristics	N (%)	
Age (years)	65-69	132 (37.5)
	70-74	96 (27.3)
	75-79	55 (15.6)
	80-84	60 (17.0)
	85-89	8 (2.3)
	90+	1 (0.3)
Gender	Male	160 (44.9)
	Female	196 (55.1)
Residency	Large urban center	152 (42.7)
	Medium/small urban center	77 (21.6)
	Rural/Northern	127 (35.7)
Education	Some high school	9 (2.5)
	High school graduate	61 (17.1)
	Trade/technical school	66 (18.5)
	Some college/university	60 (16.9)
	College/university graduate	160 (44.9)
Living arrangements	Live alone	110 (30.9)
	Live with spouse/partner/caregiver	220 (61.8)
	Live with other adults and children	23 (6.5)
	Prefer not to say	3 (0.8)
Overall health status	Excellent	22 (6.2)
	Very good	118 (33.4)
	Good	148 (42.0)
	Fair	53 (15.0)
	Poor	12 (3.4)

The mean number of symptoms was 6.2, with a range of 0 to 23. Eleven reported having had no symptoms over that time. Those with 3 or 4 symptoms accounted for 21.4%. Back pain (46.9%), joint pain (44.4%), and insomnia (38.5%) were the most frequently experienced (Table 2). Regarding colds and allergies, a seasonal effect was likely in place, given that data collection took place in late Fall/early Winter.

**Table 2.** Frequency of Minor Ailments.

Rank	Symptom	N	%
1	Back pain	167	46.9
2	Joint pain	158	44.4
3	Insomnia	137	38.5
4	Fatigue/low energy	136	38.2
5	Allergy	131	36.8
6	Peeing problems	113	31.7
7	Dry eyes	108	30.3
8	Gas	93	26.1
9	Muscle pain	92	25.8
10	Headache	89	25.0
11	Dry or itchy skin	88	24.7
12	Heartburn	84	23.6
13	Tinnitus	81	22.7
14	Dry mouth	69	19.4
15	Cold or flu	68	19.1
16	Stress	67	18.8
17	Leg cramps	66	18.5
18	Constipation	52	14.6
19	Diarrhea	46	12.9
20	Blepharitis	44	12.4
21	Ear wax	44	12.4
22	Hemorrhoids	42	11.8
23	Dizziness	35	9.8
24	Rash	32	9.0
25	Loneliness	29	8.1
26	Nail fungus	28	7.9
27	Cold sore	22	6.2
28	Nausea/vomiting	7	1.9
29	Skin infection	4	1.1
Gender specific ailments (male = 160/female = 196)			
	Erectile dysfunction	44	27.5
	Vaginal dryness	23	11.7

Joint pain emerged as the most disruptive over the recall period (3.8 on a 7-point gradient; Table 3). Nausea and vomiting, although less frequently experienced, ranked second with an average disruption score of 3.7.

**Table 3.** Disruption to Daily Activities.

Rank	Symptom (N)	Mean disruption score (SD)
1	Joint pain (158)	3.8 (1.4)
2	Nausea/vomiting (7)	3.7 (1.3)
3	Fatigue/low energy (136)	3.5 (1.6)
4	Muscle pain (92)	3.5 (1.7)
5	Stress (67)	3.5 (1.5)
6	Back pain (167)	3.5 (1.6)
7	Insomnia (137)	3.4 (1.5)
8	Diarrhea (46)	3.4 (1.7)
9	Leg cramps (66)	3.2 (1.6)
10	Cold or flu (68)	3.1 (1.6)
11	Peeing problems (113)	3.1 (1.6)
12	Headache (89)	3.0 (1.6)
13	Dizziness (35)	2.9 (1.4)
14	Rash (32)	2.8 (1.5)
15	Constipation (52)	2.7 (1.6)
16	Loneliness (29)	2.7 (1.6)
17	Heartburn (84)	2.7 (1.5)
18	Cold sore (22)	2.7 (1.8)
19	Hemorrhoids (42)	2.6 (1.5)
20	Blepharitis (44)	2.6 (1.5)
21	Allergy (131)	2.4 (1.4)
22	Dry or itchy skin (88)	2.4 (1.3)
23	Gas (93)	2.3 (1.4)
24	Tinnitus (81)	2.3 (1.3)
25	Dry eyes (108)	2.3 (1.3)
26	Nail fungus (28)	2.1 (1.4)
27	Dry mouth (69)	2.1 (1.2)
28	Earwax (44)	2.1 (1.3)
29	Skin infection (4)	1.5 (1.0)
Gender specific ailments		
	Vaginal dryness	2.4 (1.6)
	Erectile dysfunction	2.3 (1.6)

Disruption level based on a graded scale of 1 (No disruption), 2 (Minimal disruption – can be easily ignored without effort), 3 (Mild disruption – can be ignored with effort), 4 (Moderate disruption – cannot be ignored but does not influence your daily activities), 5 (Moderately severe disruption – cannot be ignored and occasionally limits your daily activities), 6 (Severe disruption – cannot be ignored and often limits your concentration on daily activities), and 7 (Very severe disruption – cannot be ignored and markedly limits your daily activities and often requires rest).

While symptoms were quantified for a 2-week period, responders were also asked to indicate symptom duration beyond that window. The vast majority exceeded 2 weeks, except for 2 (cold/flu and skin infection). Table 4 represents the responders who exceeded that time frame. For example, of the 131 who claimed allergy symptoms, 111 stated they had lasted for a month or more (with those numbers posted), while 20 stated they were limited to the recall period. Vaginal dryness was reported by women as persisting beyond 2 weeks in all 23 cases, with joint pain (98.7%) seen to a similar extent. Many responders reported symptoms lasting several years or longer.

**Table 4.** Duration of Symptoms Experienced by Individuals.

Symptom	N	1 month	3 months	6 months	1 year	Several years	>5 years
Allergy	131	2	4	6	4	29	66
Back pain	167	1	4	6	17	44	73
Blepharitis	44	0	2	2	4	12	12
Cold or flu	68	4	3	0	2	5	5
Cold sore	22	0	1	0	0	3	9
Constipation	52	1	2	3	4	13	19
Diarrhea	46	1	2	2	5	9	13
Dizziness	35	0	3	3	5	12	5
Dry eyes	108	1	3	4	10	46	33
Dry mouth	69	0	6	3	6	28	16
Dry or itchy skin	88	2	3	3	9	35	26
Earwax	44	0	0	1	4	9	22
Fatigue/low energy	136	2	6	11	17	46	31
Gas	93	2	3	5	13	29	25
Headache	89	2	1	1	3	20	28
Heartburn	84	0	1	3	8	26	31
Hemorrhoids	42	0	1	1	4	13	17
Insomnia	137	1	2	1	22	52	44
Joint pain	158	1	6	4	20	53	72
Leg cramps	66	0	2	4	12	23	17
Loneliness	29	0	2	1	2	11	10
Muscle pain	92	4	4	6	11	33	23
Nail fungus	28	0	0	0	3	18	4
Nausea/vomiting	7	0	1	0	1	2	0
Peeing problems	113	2	1	7	26	45	28
Rash	32	0	4	2	4	9	9
Skin infection	4	0	0	0	0	1	1
Stress	67	0	2	3	3	28	21
Tinnitus	81	1	0	1	9	32	34
Gender specific ailments							
Erectile dysfunction	44	0	1	2	5	15	20
Vaginal dryness	23	0	0	0	4	10	9

OTC medicines were the most common treatment choice in 16 situations, while doing nothing/watchful waiting was the main recourse in 13 (Table 5). OTC medicine use was more common for headaches (63.2%) and heartburn (61.2%), while doing nothing/watchful waiting was common for tinnitus (80.5%) and loneliness (74.2%). Turning to a herbal medicine reached peak incidence for nausea/vomiting (18.2%) and leg cramps (15.5%). Percentages can exceed 100% due to multiple interventions.

**Table 5.** Measures Undertaken by Responders to Alleviate Their Symptoms.

Symptom	Measures undertaken (%)				
	Did nothing/watchful waiting	Home remedies	Used a herbal medicine	Used an OTC medicine	Consulted a HCP
Allergy	29.4	18.1	3.1	45	4.4
Back pain	27.9	24.2	2.3	33.3	12.3
Blepharitis	22.8	28.1	8.8	29.8	10.5
Cold or flu	14.7	28.4	10.5	40	6.3
Cold sore	28.6	10.7	7.1	39.3	14.3
Constipation	27.1	11.9	11.9	40.7	8.5
Diarrhea	33.3	7.4	7.4	38.9	13
Dizziness	53.5	9.3	9.3	18.6	9.3
Dry eyes	19.5	23.3	3.8	48.9	4.5
Dry mouth	46.8	28.6	5.2	14.3	5.2
Dry or itchy skin	18.3	23.9	12.8	35.8	9.2
Earwax	44.7	27.7	6.4	12.8	8.5
Erectile dysfunction	71.7	2.2	6.5	8.7	10.9
Fatigue/low energy	45.8	23.9	7.7	14.8	7.7
Gas	60.6	7.1	9.1	19.2	4
Headache	16	13.2	3.8	63.2	3.8
Heartburn	16.3	5.1	10.2	61.2	7.1
Hemorrhoids	25.5	23.6	7.3	38.2	5.5
Insomnia	42	19.1	12.3	19.1	7.4
Joint pain	15	24.8	7.3	38.9	14.1
Leg cramps	34.5	25	15.5	17.9	7.1
Loneliness	74.2	9.7	3.2	6.5	6.5
Muscle pain	11.6	30.1	5.5	39	13.7
Nail fungus	35.5	12.9	12.9	16.1	22.6
Nausea/vomiting	36.4	9.1	18.2	36.4	0
Peeing problems	65	5	3.3	11.7	15
Rash	27	10.8	8.1	43.2	10.8
Skin infection	25	25	0	25	25
Stress	43.2	21	9.9	12.3	13.6
Tinnitus	80.5	5.7	3.4	4.6	5.7
Vaginal dryness	29.2	12.5	0	41.7	16.7

Table 6 outlines whether professional help was sought at any time beyond this recent 2-week period. The highest rates of consultation were for rash (78.1%), joint pain (76.6%), and dry eyes (73.1%). In contrast, loneliness (27.6%) and cold/flu (33.8%) were on the lowest end.

**Table 6.** Extent Responders Sought Help at Any Time.

Symptom	N	Sought help	Did not seek help	Help-seeking percent
Allergy	131	74	57	56.5
Back pain	167	107	59	64.1
Blepharitis	44	25	18	56.8
Cold or flu	68	23	45	33.8
Cold sore	22	13	9	59.1
Constipation	52	23	29	44.2
Diarrhea	46	24	22	52.2
Dizziness	35	19	16	54.3
Dry eyes	108	79	29	73.1
Dry mouth	69	28	41	40.6
Dry or itchy skin	88	46	42	52.3
Earwax	44	26	18	59.1
Erectile dysfunction	44	30	14	68.2
Fatigue/low energy	136	58	78	42.6
Gas	93	22	70	23.7
Headache	89	42	46	47.2
Heartburn	84	56	28	66.7
Hemorrhoids	42	25	17	59.5
Insomnia	137	61	76	44.5
Joint pain	158	121	37	76.6
Leg cramps	66	31	35	47.0
Loneliness	29	8	21	27.6
Muscle pain	92	51	41	55.4
Nail fungus	28	18	10	64.3
Nausea/vomiting	7	5	2	71.4
Peeing problems	113	65	48	57.5
Rash	32	25	7	78.1
Skin infection	4	2	2	50.0
Stress	67	39	28	58.2
Tinnitus	81	44	37	54.3
Vaginal dryness	23	12	11	52.2

Table 7 outlines responder satisfaction with the treatments undertaken for symptoms they had experienced. Ailments with high mean satisfaction ratings were cold sore (5.4) and headache (5.3). Table 8 depicts responder confidence in their ability to treat/manage their ailments.

**Table 7.** Satisfaction With How the Ailment Was Treated/Managed.

Rank	Symptom (N)	Treatment satisfaction mean (sd)
1	Cold Sore (22)	5.4 (1.8)
2	Headache (89)	5.3 (1.6)
3	Heartburn (84)	5.2 (1.5)
4	Hemorrhoids (42)	5.1 (1.5)
5	Earwax (44)	5.1 (1.3)
6	Constipation (52)	5.0 (1.6)
7	Cold or flu (68)	4.9 (1.8)
8	Dry eyes (108)	4.9 (1.3)
9	Leg cramps (66)	4.8 (1.7)
10	Allergy (131)	4.7 (1.9)
11	Diarrhea (46)	4.6 (1.7)
12	Skin infection (4)	4.5 (2.4)
13	Gas (93)	4.5 (1.8)
14	Dry mouth (69)	4.5 (1.7)
15	Dry or itchy skin (88)	4.4 (1.3)
16	Back pain (167)	4.4 (1.7)
17	Blepharitis (44)	4.3 (1.6)
18	Muscle pain (92)	4.3 (1.6)
19	Joint pain (158)	4.2 (1.5)
20	Dizziness (35)	4.2 (1.8)
21	Rash (32)	4.0 (1.7)
22	Stress (67)	4.0 (1.6)
23	Fatigue/low Energy (136)	3.9 (1.6)
24	Insomnia (137)	3.9 (1.8)
25	Nausea/vomiting (7)	3.7 (1.6)
26	Loneliness (29)	3.7 (1.6)
27	Peeing problems (113)	3.6 (1.8)
28	Nail fungus (28)	3.3 (1.8)
29	Tinnitus (81)	3.2 (1.8)
Gender specific ailments		
	Vaginal dryness (23)	4.7 (1.6)
	Erectile dysfunction (44)	2.9 (1.8)

Satisfaction level based on Likert scale of 1 (not satisfied), 2 (unlabeled), 3 (somewhat satisfied), 4 (unlabeled), 5 (moderately satisfied), 6 (unlabeled), and 7 (completely satisfied).

**Table 8.** Confidence in Ability to Treat/Manage the Ailment.

Rank	Symptom (N)	Treatment confidence mean (sd)
1	Cold Sore (22)	5.7 (1.7)
2	Headache (89)	5.7 (1.4)
3	Diarrhea (46)	5.5 (1.6)
4	Heartburn (84)	5.4 (1.3)
5	Constipation (52)	5.4 (1.4)
6	Dry eyes (108)	5.4 (1.3)
7	Cold or flu (68)	5.4 (1.4)
8	Allergy (131)	5.4 (1.4)
9	Hemorrhoids (42)	5.3 (1.4)
10	Earwax (44)	5.1 (1.4)
11	Dry or itchy skin (88)	5.1 (1.5)
12	Skin infection (4)	5.0 (2.3)
13	Back pain (167)	5.0 (1.4)
14	Blepharitis (44)	5.0 (1.6)
15	Gas (93)	5.0 (1.8)
16	Dry mouth (69)	4.9 (1.6)
17	Nausea/vomiting (7)	4.7 (1.6)
18	Leg cramps (66)	4.6 (1.6)
19	Muscle pain (92)	4.6 (1.6)
20	Rash (32)	4.4 (1.8)
21	Fatigue/low Energy (136)	4.4 (1.7)
22	Loneliness (29)	4.4 (1.5)
23	Joint pain (158)	4.4 (1.7)
24	Dizziness (35)	4.3 (1.5)
25	Insomnia (137)	4.2 (1.8)
26	Stress (67)	4.1 (1.6)
27	Peeing problems (113)	3.9 (1.8)
28	Nail fungus (28)	3.6 (1.9)
29	Tinnitus (81)	3.5 (1.7)
Gender specific ailments		
	Vaginal dryness (23)	4.5 (1.6)
	Erectile dysfunction (44)	3.3 (2.1)

Confidence level based on Likert scale of 1 (not confident), 2 (unlabeled), 3 (some confidence), 4 (unlabeled), 5 (moderate confidence), 6 (unlabeled), and 7 (complete confidence).

## Discussion

Minor ailments are an important component of health across the world. And yet, they often do not garner the same attention as conditions such as diabetes, cardiovascular disease, or mental health. This is understandable; the impact of those 3 alone on society is staggering. Despite their name, however, the cumulative impact of minor ailments is far from trivial. They represent the base of the symptom pyramid in terms of sheer frequency and account for many trips to primary care. In Canada, the most common reasons for visits have included colds, cough, back and joint pain, sore throat, dyspepsia, fever, headache, fatigue, and dermatitis.^10^ Others may suffer somewhat in silence (erectile dysfunction, vaginal dryness, and peeing problems). As patients age, the chances of experiencing a wider variety will of course increase.

This study sought to explore the nature and extent of minor ailments experienced by older adults, as well as treatment and behavior patterns. While previous reports have outlined how often they occur, the relevant types, and treatment choices made, the symptoms presented here were much broader. This allowed less common ones to be quantified (within the context of the others), such as hemorrhoids and cold sores. And in the same vein, ailments not considered minor (hypertension, diabetes, acute otitis media, chest pain, shortness of breath, and insomnia) were not in play.^9,10^ This was all specific to older adults and that will add to our knowledge of primary care situations.

Participants who were predominantly educated and largely in good health reported a mean of 6.2 minor ailments over a recent 2-week period. For comparison, British citizens experienced an average of 5.2 ailments over the same timeframe.^3^ Three or 4 were reported by every fifth subject. Frequently reported ailments included back and joint pain, insomnia, and fatigue/low energy. These findings align with reports on aging, which point to chronic pain, disturbed sleep, and declining energy as common complaints in late life.^30,31^ Nervousness, insomnia, colds, indigestion, pain, headache, and tiredness have been identified as common across Western Europe.^32^

The rate at which symptoms were reported can be compared to the rates seen with McAteer et al.^9^ Our study involved 356 subjects 65 years and over, drawn from a citizen’s panel, over a 2-week period, while McAteer collected data from 2474 adults aged 18 to 60 years, also with a 2-week recall, but drawn from 20 UK medical practices. The percentages for 5 symptoms were as follows (current study vs McAteer): fatigue/low energy (38.2% vs 41.3%), joint pain (44.4% vs 31.2%), headache (25.0% vs 38.7%), heartburn (23.6% vs 18.2%,) and constipation (14.6% vs 9.6%). The largest divergences (approximately 13 points) were seen with 2 of those, which might be explained by a number of factors, including age groups selected.

For further comparison, the prevalence of constipation among older adults has ranged from 16 to 32% (also depending on a number of factors).^33^ Finally as 1 last example, regarding back pain, a survey of all adults in the same province determined the prevalence to be 61.8%, in this case over a 1-year period.^34^

As expected in this population, symptoms caused some disruption to daily activities. However, our data runs contrary to reports where a more significant impact was noted.^31,35^ For perspective, with 3.8 being the highest score, it falls to the higher end between 3 (*Mild disruption – can be ignored with effort*) and 4 (*Moderate disruption – cannot be ignored but does not influence your daily activities*). The subjects in the current report may have been healthier, perhaps reflected in the numbers that claimed very good or excellent health. By comparison, McAteer et al determined that 20.4% of all those reporting joint pain (759 of 2474) in their report fell into a category of *high interference with daily life*.^9^

One influence on the extent that daily life is impacted will be the chronicity of symptoms. Most can likely attest to the sheer annoyance a cold or cough can cause for a week or 2. However, in our subjects, the vast majority of situations exceeded 2 weeks. Many responders reported symptoms lasting several years or longer, including insomnia. A review of that condition found prevalence ranges from 30% to 48% in the elderly and is associated with significant morbidity if left untreated.^36^ Our subjects reported a mean disruption of score of 3.4, which does provide some insight into its impact, although the Insomnia Severity Index would have been a better measure (had this symptom been our sole focus).^37^ Less than 10% consulted with a healthcare provider for this issue within our 2-week window, while 44.5% had sought help at some point in the past.

Patients likely have a ‘break point’ as to when it is acceptable to seek care, perhaps balancing duration/severity against not wanting to trouble a doctor for what might be self-perceived as a trivial complaint.^38^ The lay care responses of 667 older individuals provide further insight.^39^ These individuals recorded experiences with 26 common symptoms over a 3-week period, some minor and some serious. Their responses to the minor symptoms did not align with expectations for seeking professional care. A significant proportion faced potential risks associated with at least 1 symptom during this time frame. It appeared older individuals often underestimate the seriousness of symptoms that clinicians identified as potentially risky. Conversely, in a survey of Canadians of all ages with a headache, cold, or heartburn, 16% made an appointment with a doctor even though they reported low symptom severity.^6^

Responders were asked what they did (if anything over 2 weeks) for the symptoms they experienced, then how satisfied they were as a result of the approach they took (7-point scale). This was therefore an evaluation of any or all modalities chosen by the patient, from using an OTC medicine, care from a provider, or even the choice to do nothing (wait-and-see). Isolating 3 for comparative purposes, heartburn garnered a 5.2, while joint pain was 1 unit less at 4.2, with tinnitus another further unit less (3.2), encompassing a range from *moderately satisfied* down to *somewhat satisfied*.

This patient-centered outcome has received a lot of attention in the literature. Tools to measure overall treatment satisfaction have been developed^40^ and satisfaction has been examined for a wide array of situations including constipation,^41^ insomnia,^42^ dry eye disease,^43^ and low back pain.^44^

A systematic review evaluated self-care interventions of community-dwelling older adults, focusing on physical and psychological health outcomes.^45^ Results emphasize that satisfaction is not solely dependent on the treatment modality, but also on individual expectations and experiences. Some individuals may be satisfied with a wait-and-see approach, while others may be disappointed with the results of OTC medication. Satisfaction levels reflect the perceived effectiveness of treatments and the ease of managing symptoms, shaping individual confidence in addressing similar ailments in the future.

Overall, patients tend to be satisfied with the OTC agents they use, often rooted in personal experience and familiarity.^46,47^ A study in 1 Canadian city explored public perceptions of these medicines by assessing 15 common categories across 3 dimensions: effectiveness, safety, and familiarity.^48^ Findings showed moderate perceptions of effectiveness (ranging from 5.1 to 7.3 on a 10-point scale). In the same Canadian city, respondents reported using an average of 2.7 OTC products over 6 months and expressed high satisfaction with product effectiveness, particularly for pain relievers and stomach remedies.^49^

A key consideration for this report was which ailments to include. While definitions of minor ailments exist,^25,50,51^ there is debate as to which ones are in fact ‘minor’. There was no precedence to follow in creating a list where *the* most common ailments were included, with each 1 meeting the precise criteria for what is minor. Instead, a list that reasonably reflected an array of common minor conditions was the goal. It was not designed to determine, from a complete list of all possible minor ailments, a ranking of most to least common. Table 2 therefore does not necessarily reflect *the most common* ailments, but rather the prevalence of the 30 presented to each gender for consideration.

Regarding the number of symptoms to include, choosing 10 or 20 would have reduced responder burden dramatically, a legitimate consideration for such surveys. A list of 10 would have meant situations like dry eyes (30.3%) and beyond would not have been captured. Resorting to 20 would remove ones like hemorrhoids (9.8%) and loneliness (8.1%). United Kingdom researchers investigated 25 symptoms, but included potentially serious ones,^5^ as did Stoller’s list.^39^ Either way, the resultant list of 30 might provide future researchers with better insight into a list for further study.

When a person acquires a minor ailment, a decision will be made on how to address it. Depending on severity, familiarity, and a host of other factors, a measure or measure(s) is adopted, ranging from simply observing it for a while, using some form of medicine, or seeking professional care. Such decisions can be easy to make, or rather daunting, again for the same reasons. Success in navigating self-care situations has been attributed to 7 pillars: health literacy, self-awareness, physical activity, optimal use of products and services, healthy eating, risk avoidance, and good hygiene.^51^ Silva et al examined the impact of socioeconomic factors when adopting self-care as a treatment modality for various conditions (including influenza, pain disorders, allergic rhinitis, dyspepsia), noting that education, income, lifestyle, and residential segregation were possible determinants.^52^ Others report that age over 75 years and separation from a spouse can negatively impact decision-making.^53^

Once enacted, the person will assess its value in a return to health. Our study found varying confidence levels among older adults in managing minor ailments. Individuals exhibited the highest confidence when managing conditions such as cold sores, headaches, and diarrhea, which are commonly self-managed or treated with OTC medications. In contrast were lower confidence levels observed for erectile dysfunction, tinnitus, and nail fungus, where individuals may feel less certain about self-care and more inclined to seek professional help.

Higher confidence in managing minor ailments may suggest that individuals are more likely to choose self-care strategies. Factors contributing to confidence will likely include knowing what agents are even available for symptom relief, health literacy, symptom severity, and of course self-efficacy relative to any given situation.^54^ A study explored the attitudes and behaviors of consumers regarding self-care of minor ailments in England and Wales.^55^ A key finding was that prior successful experiences with self-care significantly increased consumer confidence, with 84% who had previously self-treated opting to do so again for new episodes. Almost half attempted self-treatment for new ailments.

A cross-sectional survey aimed to quantify the level of confidence in knowing when patients should seek medical care for 21 health-related situations.^56^ The findings revealed that public confidence in deciding when to seek medical care varied widely by condition, with notably lower confidence for minor ailments such as tension headaches, tinea corporis, constipation, and low back pain.

All the symptoms experienced by responders in our report describe approaches similar to other reports – varying degrees of doing nothing, non-pharmacological measures, OTC medicines, and professional care. It might be a rare situation where a person only attempts 1 measure for a given problem. For example, a person navigating a tension headache could do nothing for the first few hours, then close their eyes for a few minutes, add a warm forehead compress, before taking some ibuprofen, which would qualify as 3 modalities from our list of options.

In our case, the importance of OTC medicines for minor ailments was evident. They were the most common choice in 16 situations and were common for headaches, heartburn, and dry eyes. Doing nothing (watchful waiting) was still very prevalent for these situations, followed by some form of a home remedy. While the extent will vary from study to study, and illness to illness, our data generally falls in line with other reports. Self-medication with OTC medicines is common in self-care.^57^ A recent review of self-medication behaviors across 4 continents reported an overall prevalence of 67%.^58^ A study of American older adults found that 76% used OTC medications.^59^ A multi-national survey of self-care behaviors among adults in the UK, USA, Australia, and Japan found that the overall prevalence of self-care was highest in Japan (54.9%) and around 40% in the other countries.^60^ The most common form of self-care across all 4 nations was the use of supplements, which often overlap with OTC products. A UK population-based study highlighted the management of symptoms, where nearly half of reported symptoms led to inaction, and lay care (primarily OTC medicine) was used in 35% of cases.^5^

Stoller et al found a range of OTC medicine use in older adults from 76.0% for headache to 0% for fainting.^39^ Another report focused on how older adults manage daily symptoms by examining their use of self-care practices and medical care.^61^ The study found that nearly all participants (99%) experienced at least 1 symptom during the follow-up period. Home remedies were used by 32.6% of participants, especially for upper respiratory symptoms. Rest was the most common self-care practice, with 86.5% engaging in it on about half of the days they experienced symptoms. Almost 3-quarters of participants reduced their activities during illness. OTC medicines were used by 46.1%. Around a quarter sought medical care from a physician, with musculoskeletal symptoms prompting the highest number of visits. There were gender differences in all these approaches.

A study on self-medication in South Australia’s older adult population found that product use (OTC and alternative medicines) ranged from 17.7% to 35.5%.^62^ Some of the more common were vitamins and minerals, herbal medicines, nutritional supplements, analgesics, laxatives, and low-dose aspirin. Participants seemed to self-medicate appropriately (based on approved indications), suggesting they were informed consumers, but the authors raised concerns about the risk of analgesics and aspirin.

A UK-based study examined how people 60 years and over utilize OTC medicines.^63^ Use of these products appeared to be diverse and often driven by hope, rather than scientific evidence or knowledge of them. The authors suggested that older adults, much like other age groups, are increasingly engaging in health consumerism, reflecting a broader societal trend of commodifying health and self-care.

Regarding study limitations, the survey attempted to distill complex human health behavior down to numerical values, with all the limitations that are applicable to that process. Second, the sample may not have been a true reflection of older adults in the province. Sensitive topics such as erectile dysfunction and vaginal dryness may have been under-reported. Also, our data may have captured healthier and more educated subjects. Countering that, however, is that the percentage who completed college or university closely mirrors national statistics.^64^ A slight majority of participants resided in urban areas, which again aligns with population numbers. Regarding overall health, the majority reported good or very good health (41.6% and 33.1%, respectively), which reflects a Canadian study on individuals aged 65 years and older, where 75.6% reported good health.^65^

A third limitation is the reliance on self-report and whether a responder accurately portrayed the symptom (condition) they claimed to have, as this was not verified.

The use of an online survey could have introduced selection bias, as it may exclude older adults who have limited familiarity with digital technologies. As a result, the sample may disproportionately represent more educated, urban-dwelling, or technologically literate individuals, which could limit the generalizability of the findings to the broader older adult population in Saskatchewan, particularly those from rural or underserved areas.

Another limitation is that the survey provides a snapshot of experiences and behaviors at a single point in time, which will affect seasonal conditions. A 2-week window in late Fall/early Winter meant acute allergic rhinitis was starting to wane, while colds and flu were more in play. Due to lower humidity at that time of year, dry eyes and dry skin may have also been more prevalent. For upper respiratory infections, the Center for Disease Control has reported on who experienced a cold over a 2-week period.^66^ The percentages (all ages) who reported a cold was 16.6% for January to March, 8.5% in April to June, 7.0% in July to September, and 13.7% in October to December. Across all time periods, colds were more common in younger people than those older. We reported colds or flu at 19.1%. A national survey of Canadians noted that 6% to 16% of older adults claimed to have had a cold or flu in April.^6^

While the list of ailments was refined through a combination of literature review and expert opinion, it may not fully represent an appropriate list of common minor health issues experienced by older adults. Finally, the options made available to responders for each symptom, such as treatment choices they undertook, symptom duration, and the level of disruption to daily activities, may not have accurately reflected all possible options.

## Conclusion

This study sheds light on the impact of minor ailments amongst older adults. It is unique in the breadth of symptoms examined, all done from the perspective where serious ones like shortness of breath, heart palpitations, and unintentional weight loss were excluded. Musculoskeletal pain, fatigue, insomnia urinary problems, and dry eyes highlight the diverse nature of ailments, and were often of a chronic nature. There was a tendency for proactive self-care, particularly in the use of OTC medications. These older adults seemed to demonstrate some confidence in managing their minor health issues. OTC medicine played an important role in getting back to health, but doing nothing/watchful waiting also was a prominent course of action. In light of these findings, healthcare providers should continue to recognize minor ailments as an integral aspect of geriatric care. Gaps in our knowledge would be aided by future longitudinal studies to follow individuals dealing with minor ailments as they unfold. The findings for treatment satisfaction and confidence may now provide potential baseline measures as the public navigates self-care situations.
